# Meat–Egg–Dairy Consumption and Frailty among Chinese Older Adults: Exploring Rural/Urban and Gender Differences

**DOI:** 10.3390/nu16091334

**Published:** 2024-04-28

**Authors:** Keqing Zhang, Jiangcheng Wu

**Affiliations:** 1 School of English and International Studies, Beijing Foreign Studies University, Beijing 10089, China; zhangkeqing@bfsu.edu.cn; 2 School of Japanese and International Studies, Beijing Foreign Studies University, Beijing 10089, China

**Keywords:** frailty, MED consumption, Chinese Longitudinal Healthy Longevity Survey, rural/urban differences, gender differences

## Abstract

The dietary patterns of older adults, particularly in relation to meat, egg, and dairy (MED) consumption, significantly impact frailty, a state of heightened vulnerability to adverse health outcomes. This paper investigates the association between MED consumption and frailty among older Chinese adults, considering rural/urban disparities and gender differences. Analyzing data from the Chinese Longitudinal Healthy Longevity Survey (CLHLS) spanning from 2008 to 2018, this study explores how MED consumption influences frailty levels over time. The results show that moderate MED consumption is associated with slower frailty progression, suggesting a protective effect against frailty among older adults. However, excessive MED consumption, particularly among rural residents and females, is linked to accelerated frailty progression. Urban residents and males report higher MED consumption levels, possibly due to their greater access to diverse food options and traditional dietary preferences. The findings underscore the complex interplay between dietary habits, demographic factors, and frailty outcomes. Understanding these dynamics is crucial for developing targeted interventions to mitigate frailty risk factors and promote healthy aging among Chinese older adults.

## 1. Introduction

The dietary patterns of older adults play a vital role in determining their health and well-being, particularly in the context of frailty, which represents a state of increased vulnerability to adverse health outcomes [1]. As the global population ages, understanding the intricate relationship between dietary patterns and frailty becomes increasingly important for promoting healthy aging and reducing the burden of age-related health issues. Frailty, characterized by decreased physiological reserve and increased susceptibility to stressors, represents a significant health concern among older adults. Individuals with higher frailty levels are at heightened risk of functional decline, disability, hospitalization, and mortality [1,2]. While frailty can be influenced by a variety of factors, including genetics, chronic diseases, physical inactivity, and social isolation, emerging evidence suggested that dietary factors play an important role in the development and progression of frailty among older adults. Understanding the impact of specific dietary components on frailty is of great interest to researchers and healthcare practitioners.

Compared with other dietary components, research specifically exploring the association between meat, egg, and dairy (MED) products consumption and frailty among older adults is rather limited. Recommended by the WHO, meat, eggs, and dairy products are valuable sources of protein, which is essential for maintaining overall health and well-being, especially meeting the dietary requirement of specific population groups such as children and older adults. Some studies have revealed the crucial role of protein in preserving muscle mass, strength, and function, while the excessive consumption of certain MED items, such as red and processed meats, has been linked to adverse health outcomes, including inflammation, oxidative stress, and chronic diseases [3,4]. Research specifically focusing on MED consumption and frailty among older adults remains lacking, and this paper aims to examine such associations.

Understanding the MED–frailty relationship is particularly salient in the context of China, due to the country’s rapidly aging population and evolving dietary habits. China has been undergoing significant socioeconomic changes, with urbanization and economic development influencing dietary patterns among older adults [5]. Traditional Chinese diets, characterized by a balance of plant-based foods and modest or little amounts of animal-based products, are transitioning toward Westernized dietary patterns, characterized by increased consumption of processed foods, sugary beverages, and high-fat, high-sugar snacks [6]. With the increasing importance that the government attached to MED consumption in *The Chinese Dietary Guideline 2022* [7], the time-varying changes in MED consumption as well as frailty levels among older adults should be elucidated.

Moreover, the MED–frailty association may differ by important demographic factors such as *hukou* (household registration) status. China’s unique hukou system (household registration) required that each citizen should register in one and only one place of permanent residence. Despite increasing population migration, most people still stayed and remained where they were born, raised, and registered their *hukou* in China for most of their lives. In China, rural area residents often exhibited traditional dietary patterns characterized by a higher consumption of plant-based foods and lower intake of animal-based products compared to urban areas, indicating lower MED consumption [6]. Besides rural/urban differences, gender is another important factor to consider in the Chinese cultural context due to significant differences in gender roles. Studies have suggested that older Chinese women may have a lower consumption of animal-based products, including meat, eggs, and dairy products compared to men. Additionally, gender disparities in frailty prevalence have been observed, with women generally exhibiting higher frailty rates compared to men [8,9]. Understanding how *hukou* status and gender influence the MED–frailty relationship can inform targeted interventions to address frailty risk factors among older adults in China.

## 2. Background

### 2.1. Dietary Patterns and Frailty

Older adults often exhibit unique dietary patterns, influenced by a myriad of factors, including cultural norms, socioeconomic status, health status, and individual preferences. While dietary needs may vary among older adults, several key dietary components have been consistently associated with health outcomes in this population. For instance, the adequate intake of protein, vitamins, minerals, and fibers is essential for maintaining muscle mass, bone density, cognitive function, and overall well-being [10]. Conversely, poor dietary habits, such as excessive consumption of processed food high in sugar, salt, and unhealthy fats can contribute to the development of chronic disease and frailty among older adults [10].

Research on dietary habits and frailty in older adults mostly has focused on the Mediterranean diet [11], a popular dietary pattern in developed countries which referred to food high in fruits, vegetables, legumes, nuts, cereals, fish, and olive oil but low in meat and dairy products [12]. Using the Mediterranean diet as a prototype, dietary habits were often categorized into meat-based and plant-based patterns, and plant-based diets were found to be more effective in reducing physical frailty risk among older adults [12,13]. A longitudinal study by Chan et al. [14] examined the dietary patterns and their association with older adults’ frailty over a 5-year follow-up and found that adherence to a Mediterranean diet was associated with a lower risk of frailty development among older adults. Expanding upon the Mediterranean diet, recent research has explored distinctions among various plant-based diets or investigated specific types of healthful plants [13,15]. Other dietary patterns such as Healthy Eating Index (HEI) and Dietary Approaches to Stop Hypertension (DASH) have been identified as significant indicators of older adults’ frailty [16,17].

While the existing literature has provided valuable insights into the association between overall dietary pattern and frailty among older adults, further investigation into the specific components of these diets is warranted and lacking, in particular individual food groups such as meat, eggs, and dairy products.

### 2.2. Meat, Egg, and Dairy Product Consumption (MED) and Frailty

As a distinctive Chinese term, MED (ròu dàn nǎi, 肉蛋奶) items encompass a wide range of foods, including red and processed meats, poultry, eggs, milk, cheese, and yogurt. These foods are rich sources of essential nutrients, including high-quality protein, vitamins (such as B vitamins), minerals (such as calcium), and bioactive compounds (such as omega-3 fatty acids), which are vital for maintaining health and function in older adults [3]. While studies specifically examining the association between MED consumption and frailty are limited, some research has suggested potential benefits of some particular MED items for frailty prevention. For instance, studies investigating the association between meat consumption and frailty among older adults have yielded mixed findings. A prospective cohort study found that higher consumption of red and processed meats was associated with an increased risk of frailty among older adults [18]. Meanwhile, concerns have been raised about the impact of egg consumption on cardiovascular diseases due to their cholesterol content [19], but a prospective cohort study by Kim et al. [20] found that higher egg intake was associated with a reduced risk of frailty among older Korean adults. Moreover, although the consumption of dairy products has been linked to various health benefits, including improved bone health and reduced risk of osteoporosis [21,22], the association between dairy consumption and frailty among older adults remains understudied.

The Food and Agriculture Organization of the United Nations recognizes the importance of consuming a balanced diet that includes moderate amounts of meat, eggs, and dairy products as part of a healthy lifestyle. While the UN does not advocate for the consumption of these foods in isolation, it acknowledges their nutritional value and recommends the inclusion of all three items as part of a divers and nutritious diet [23]. Understanding the combined effects of these items on frailty necessitated a comprehensive approach that considers the interactions between different dietary components. In this study, the collective effects of three MED items will be examined simultaneously and comprehensively.

### 2.3. The Context of China: Differences by Hukou Status and Gender

Understanding the relationship between MED consumption and frailty among older adults requires a consideration of cultural, dietary, and lifestyle factors that may vary across different populations. As such, examining this association within the specific context of China provides valuable insights into the unique dietary patterns and health outcomes observed in this population, offering a distinct perspective on MED consumption and its implications for frailty risk among older adults.

The dietary patterns of Chinese older adults could be influenced by a combination of cultural traditions, economic development, and urbanization. Traditional Chinese dietary patterns, characterized by a high intake of plant-based foods such as rice, vegetables, and legumes, have gradually shifted toward a more Westernized diet with increased consumption of animal-based foods, including meat, eggs, and dairy products [6]. These dietary transitions have implications for health outcomes among Chinese older adults, with potential impacts on chronic disease risk, nutritional status, and frailty levels. Findings examining the association between dietary patterns and frailty among Chinese older adults have yielded mixed effects, highlighting the complexity of such relationship. For instance, the positive effects of certain dietary components, such as higher intake of fruits, vegetables, and whole grains, on frailty among older adults were substantiated and supported by various longitudinal studies [9,24,25]. Conversely, some other studies have identified the potential risk factors associated with frailty, including excessive consumption of processed food, and high-fat, high-sugar foods [26]. Unhealthy dietary habits characterized by a low intake of essential nutrients, such as protein and vitamins, may contribute to frailty development and exacerbate age-related declines in physical function and cognitive health [10]. While research specific to Chinese populations is limited, the existing evidence suggests the potential benefits of certain MED items for frailty prevention. From the 1990s until the introduction of new guidelines emphasizing diversity in recent years [27], meat, eggs, and milk had been considered as the primary sources of protein, with official health guidelines in China strongly advocating for a diet centered around these foods [28,29]. Whether excessive protein intake influences the frailty of older adults in China therefore gains its significance.

Moreover, understanding the MED–frailty association among Chinese older adults requires a consideration of various demographic and geographic factors that shape dietary patterns and health outcomes. As such, exploring potential rural/urban and gender differences in this relationship offers valuable insights into the nuanced interplay. Studies have shown that rural older adults in China tend to have a lower consumption of animal-based foods, including meat, eggs, and dairy products, compared to their urban counterparts [30]. This disparity in dietary patterns may be attributed to factors such as limited access to markets, lower socioeconomic status, and adherence to traditional dietary practices in rural areas. As a result, rural older adults may have different MED consumption patterns that could impact their frailty risk. Additionally, studies have shown that men and women in China may have distinct dietary preferences and consumption patterns, influenced by cultural traditions and social norms [31,32]. For example, men in China tend to have a higher consumption of meat products, including red meat and processed meats, compared to women, who may consume more plant-based foods such as vegetables and legumes. However, differences in MED–frailty associations within these groups have not been explicitly explored.

Taken together, this study aims to explore how dietary patterns, specifically MED consumption, may influence frailty among older adults in China, and discuss rural/urban as well as gender differences. Our findings will enrich the literature by focusing on the largest aging population embedded in a unique historical, cultural, and social context, seeking to provide insights into the complex interplay between diet, lifestyle, and frailty outcomes among Chinese older adults.

## 3. Method

### 3.1. Data

This study is based on data from the Chinese Longitudinal Healthy Longevity Survey (CLHLS). Aiming to investigate the determinants of healthy longevity among Chinese older adults and to understand the aging process in the population, the survey was first carried out in 22 provinces and a multi-stage non-equal target random sampling method was adopted. Moreover, to ensure an adequate sample size of older adults, the study oversampled individuals aged 80 and above. The baseline survey started in 1998, and then follow-up surveys were conducted in 2000, 2002, 2005, 2008, 2011, 2014, and 2018, respectively. All data were collected using face-to-face interviews by trained interviewers. The study was approved by the Ethical Review Committee of Peking University, and all participants signed written informed consent forms (IRB00001052–24713074).

### 3.2. Study Sample

To foster a reliable longitudinal model, this study utilized data from 2008 to 2018, and included respondents who were in all four waves. Moreover, the study sample was restricted to respondents living in the community and aged 55 years and above. Meanwhile, those with no MED consumption or frailty data were excluded, and the final study sample included 2312 respondents. For missing data on demographic characteristics and other covariates, multiple imputations were carried out.

### 3.3. Measures

#### 3.3.1. Dependent Variable

Using CLHLS data, a 39-item frailty index (FI) was created in reference to several validated methods [25,33]. These items were chosen considering their relevance to individual physical health and tendency to become more common with age. Meanwhile, such items would not be universally present among all older adults; for instance, chronic diseases and activity of daily living (ADL) difficulties. Detailed items and codes are presented in Supplementary Materials (Table S1): About half of the items were dichotomous, with responses recoded as either yes (1) or no (1). For items with ordinal responses (e.g., always, often, sometimes, seldom, and never), scores of 0, 0.25, 0.5, 0.75, and 1 were assigned to ensure they fit in the 0–1 range. The average score of the 39 items was computed with a higher score reflecting higher levels of frailty. Preliminary analysis showed that the population baseline frailty ranged from 0 to 0.603, with an average of 0.076.

#### 3.3.2. Main Independent Variable (IV)

To determine the meat–egg–dairy (MED) consumption pattern across waves, three dummy variables were created to represent whether an individual consumed meat, egg, or dairy regularly. The frequency of consumption was assessed based on China Nutrition Guidelines: for those who consumed MED every day or a few times a week, they were assigned the value of 1 (yes); otherwise, they were assigned the value 0 (no). Since the dietary patterns of some older adults may include partial vegetarianism, it was important to account for their dietary preferences: we also included fish consumption, meaning that if an individual consumes either fish or meat, the corresponding dummy variable is assigned a value of 1. The overall consumption score was calculated based on the scores of three items, ranging from 0 to 3, with a higher score indicating a greater intake of the MED items (hereafter referred to as 0-/1-/2-/3-MED consumption).

#### 3.3.3. Covariates

Sociodemographic covariates were age (in years), gender (male = 1), marital status (married/partnered = 1), *hukou* status (rural = 1), education (literacy = 1), and sufficient income for daily costs (yes = 1). Health and health behavior controls included smoking (current/past smoker = 1), drinking (current/past drinker = 1), exercise (everyday/several times a week = 1), levels of life satisfaction (0–4, with higher scores reflecting higher levels), and psychological well-being (0–4, with higher scores reflecting more positive mentality using the CESD Depression Scale).

### 3.4. Analysis

**Figure 1:**
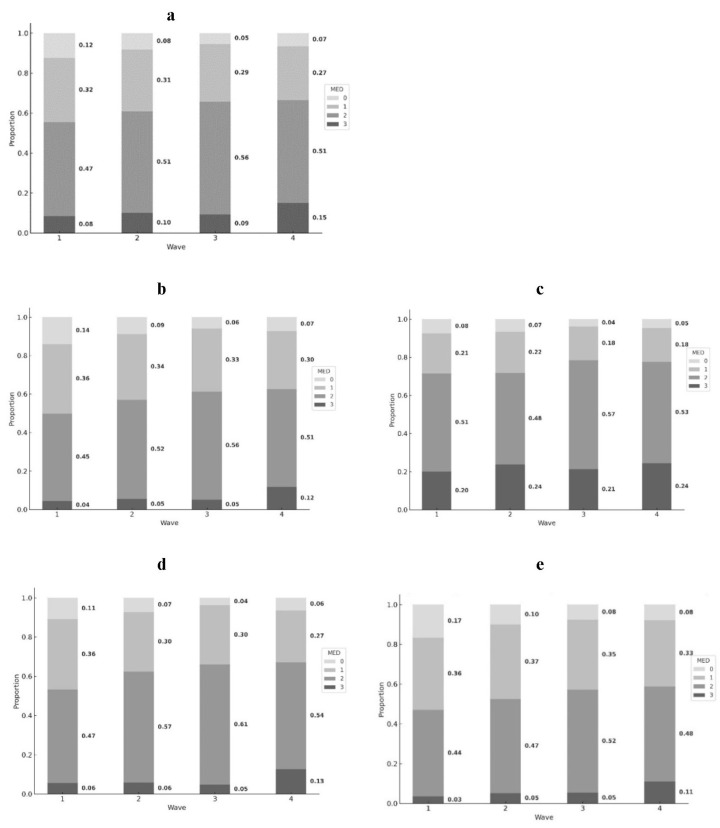
Proportions of MED consumption over four waves ((**a**) whole sample, (**b**) rural subsample, (**c**) urban subsample, (**d**) male subsample, (**e**) female subsample).

**Figure 2:**
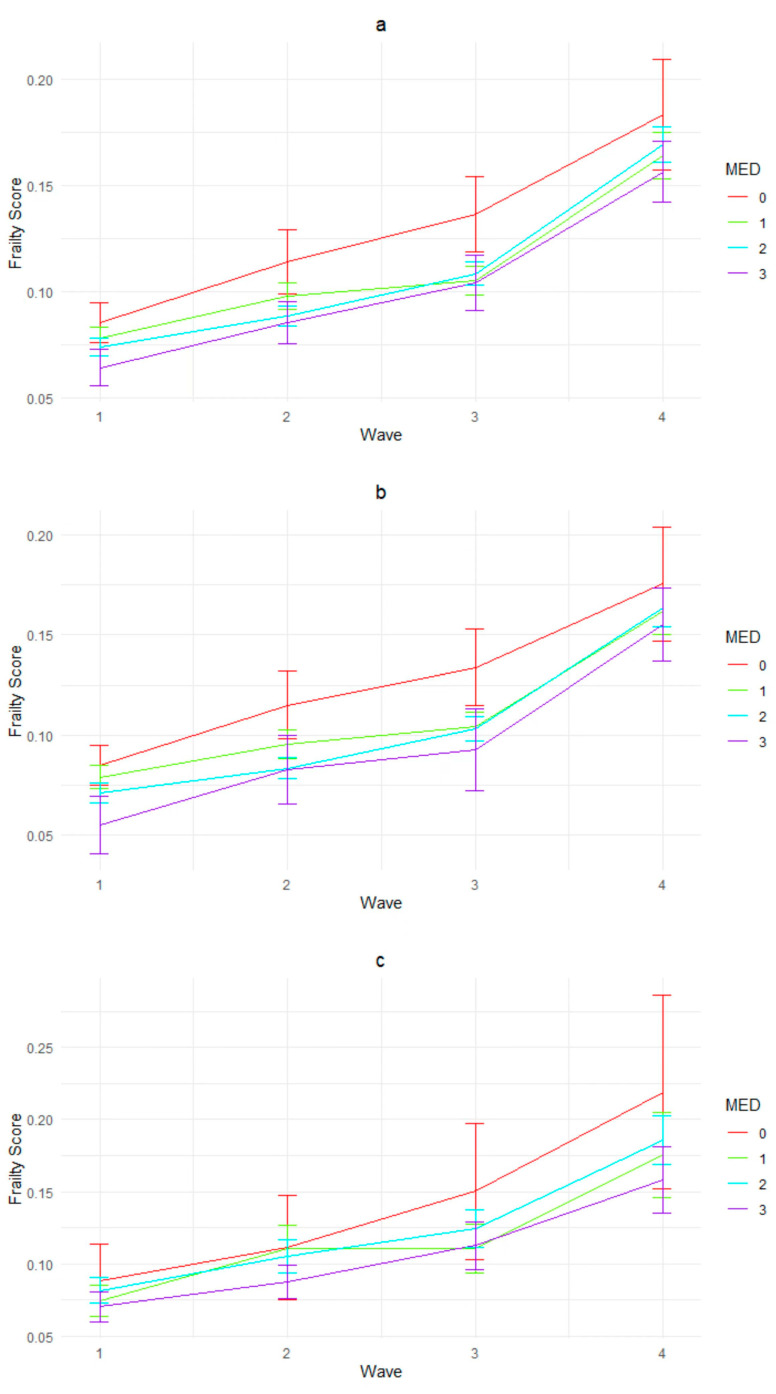

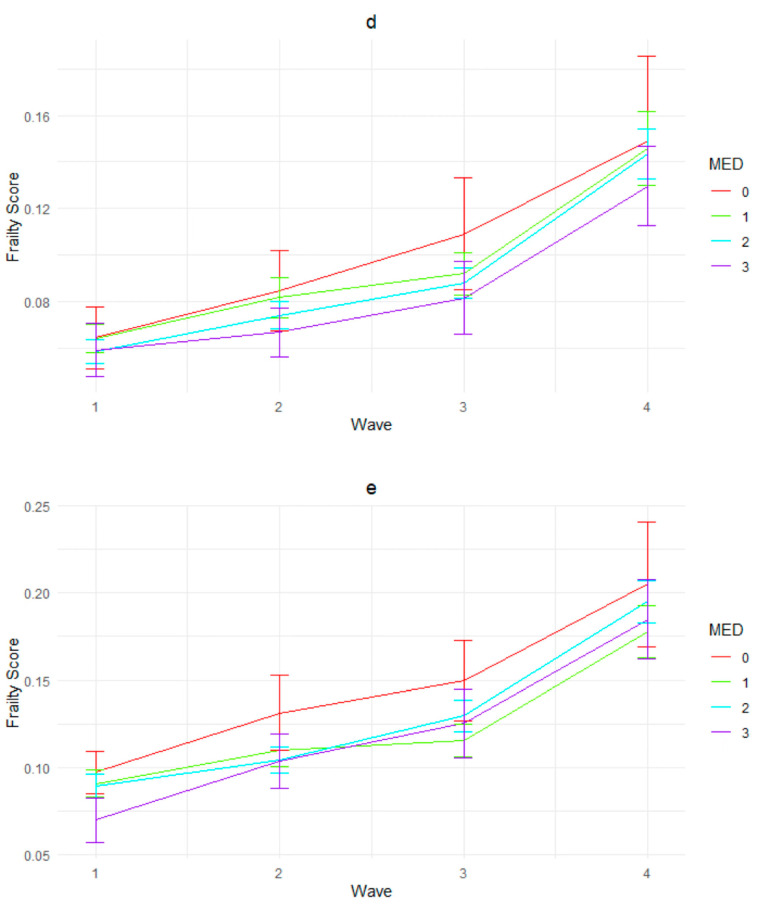
Frailty levels over four waves by MED consumption ((**a**) whole sample, (**b**) rural subsample, (**c**) urban subsample, (**d**) male subsample, (**e**) female subsample).

Data analysis was conducted using RStudio (Build 494). Descriptive statistics were summarized in Table 1 using baseline (year 2008) data. Paired *t*-tests and ANOVA tests were carried out to evaluate the rural/urban as well as gender differences across all variables. Figure 1 visualized the MED consumption proportion changes across the four waves for the whole sample (Figure 1a) as well as for the subsamples (Figure 1b–e). Moreover, Figure 2 visualized the frailty levels of older adults from different MED groups across four waves: Figure 2a was about the whole sample, while Figure 2b–e showed subsample results. To test the dynamic association between four waves of MED consumption and frailty levels, generalized estimating equations (GEEs) were adopted, a modelling approach that focuses on the population-averaged effects. Considering the fact that frailty levels ranged between 0 and 1 and the coefficients for certain important variables could be minimal, we used frailty *10 as the dependent variable in GEE models to amplify the coefficients for easier interpretation. The results are presented in Table 1, controlling for socio-demographics and health variables. Model 1 shows the results for the whole sample, and Models 2–5 include subsample variances, namely rural/urban, male/female.

## 4. Results

Sample characteristics at baseline are presented in Table 1. Overall, the Chinese older adults had a fairly high level of MED consumption: only 12.37% reported no consumption of MED products. Nearly three-quarters of them had rural *hukou* status, and there were more female respondents. More than half were literate, and most (77.64%) rated their family income as sufficient for daily living. Moreover, they scored 3.62 out of 4 for life satisfaction, and 2.95 out of 5 for psychological well-being. Baseline frailty levels ranged between 0 and 0.62, and the population average was 0.076, indicating rather low levels.

The rural–urban comparison revealed the significant advantaged nutrition status of urban residents: they reported comparatively higher proportions of 2-MED and 3-MED consumption. The advantages even extended to literacy, income levels, and exercise. The urban subsample also scored higher on life satisfaction and psychological well-being. An interesting finding lies in the fact that, although the maximum frailty level was visibly lower (0.47 in comparison to 0.60), the average subsample frailty of urban older adults was significantly higher. The same pattern was also observed in the male–female comparison: females had lower upper limits, but much higher average frailty scores. Meanwhile, the females also reported significantly lower mean MED consumption, as well as nearly all sociodemographic covariates. They had lower levels of life satisfaction but higher psychological well-being scores.

Figure 1 shows the dynamic changes in MED consumption proportions across ten years. Overall, the whole study sample experienced steady increases in 2-MED and 3-MED consumption levels over the four waves, and similar trends could be found among all four subsamples below (Figure 1b–e). However, the urban sample reported much higher proportions of 2- and 3-MED consumption compared with their rural counterparts, especially 3-MED consumption. Lastly, the reported lower mean MED consumption for females persisted across all waves.

Figure 2 visualized the dynamic relationship between MED consumption and mean frailty level across four waves. For the whole sample (Figure 2a), overall, the frailty levels increased with years, and the starting frailty met the expectation: 0 > 1 > 2 > 3-MED consumption. However, the transition from wave 2 to 3 witnessed a change, and the order remained as 0 > 2 >1 > 3-MED consumption until wave 4. The rural/urban comparison (Figure 2b,c) shows that the urban subsample has wider frailty ranges across four waves, and both ended up with 0 > 2 > 1 > 3-MED consumption. Moreover, the urban sample experienced two intersections of 2- and 3- -MED consumption, indicating that they were more susceptible to dietary changes. Gender subgroups (Figure 2d,e) had a different pattern: the females had wider frailty ranges, and the starting frailty indicated that 1- and 2-MED consumption had similar influences. Moreover, after ten years, the subsample frailty level order was 0 > 2 > 3 > 1-MED consumption, with older adults consuming only one MED item having the lowest frailty level. The males had narrower ranges, and the corresponding MED consumption for frailty order remained constant: 0 > 1 > 2 > 3.

GEE model results are presented in Table 2, in which Model 1 shows the results for the whole sample, and Models 2–5 include subsample variances, namely rural/urban and male/female. In Model 1, on average, individuals with 1-MED consumption experienced a significant decrease in frailty level by 0.052 units (compared with the reference group of 0-MED consumption). A similar decreasing effect was also detected among all subgroups. However, 2-MED consumption was only significant for female respondents, and the association was in the expected negative direction: β = −0.024. And lastly, it is worth noting that, for the rural and female residents, reporting 3-MED consumption increased the frailty level by 0.102 and 0.049, respectively, compared with the reference group, and the increase was significant.

## 5. Discussion

Focusing on older adults in China, this study examined the longitudinal associations between MED consumption and frailty level in later life and explored rural/urban and gender differences in the associations. Overall, the baseline data analysis and MED consumption trend analysis showed that older adults in China had a comparatively high level of MED consumption, while the male and urban respondents reported significantly higher levels, and such advantages persisted across four waves. This is in accordance with the previous literature, according to which males often were traditionally associated with higher protein intake and larger portion sizes, including more meat consumption, and such difference would persist into old age [34,35]. Apart from that, we speculate that differences in health consciousness and nutrition knowledge may have also contributed to this: Males may prioritize taste and satiety over nutritional considerations, while females, on the other hand, may be more likely to be health-conscious and aware of the potential health risks associated with excessive MED consumption and have a more moderate intake [36,37]. In this sense, food preferences and taste preferences may lead males to prefer protein-rich food while females may prefer lighter or plant-based options in China [38,39]. The economic empowerment and decision-making power of males within households could often lead to them prioritizing individual preferences, including higher consumption of MED products. Moreover, for Chinese older adults who experienced the great famine in 1960s, the preference for meat may be especially stronger for males [40].

As for urban/rural differences, urban residents typically had greater access to diverse food options, which may offer a wider variety of MED products, and such increased availability could contribute to higher consumption levels [41,42]. Conversely, rural areas may have limited access to fresh produce and animal products, leading to lower levels of MED consumption [43]. Moreover, we conjecture that urban, older adults often had higher socioeconomic statuses, which could afford them greater purchasing power and access to a wider range of foods, while their rural peers may have had lower incomes/pensions and face economic constraints that limited their access to more diverse MED products, including meat, eggs, and dairy products [44,45]. While a diet rich in proteins and fats could provide essential nutrients, excessive consumption, especially of processed meats high in saturated fats, could contribute to adverse health outcomes such as frailty among the urban older adults [46], while for rural residents who maintained more traditional lifestyles, their diets may include fewer processed foods and more locally sourced options, and they might also engage in more physical labor, which could contribute to better physical function and lower frailty levels [44,47,48].

The findings found in Figure 2 and Table 2 reveal the complex relationship between diet and frailty outcomes. On the one hand, they indicate that, for the whole sample and for all subgroups, the frailty levels of older adults increased consistently over the four waves, regardless of their MED consumption levels. This reflected a well-documented trend observed in aging populations globally: with advancing age, individuals experience a gradual decline in physiological function and reserve capacity across multiple organ systems, and such cumulative effects of aging and decline in functional capacity is inevitable, contributing to the progression of frailty [1,49]. This has underscored the multifactorial nature of frailty, influenced by a combination of biological, social, and environmental factors, and indicated that dietary factors alone may not be sufficient to prevent or reverse the progression of frailty among older adults [50,51]. However, the general observation that higher MED consumption was associated with lower frailty levels also suggested a potential long-term protective effect of MED consumption against frailty among older adults [14].

The longitudinal analysis utilizing GEE models revealed a notable trend wherein older adults consuming 1–2 kinds of MED items exhibited a slower increase in frailty levels compared to those with 0-MED consumption. This has suggested a potential protective effect of moderate MED consumption against frailty progression among Chinese older adults. One possible interpretation is that a moderate consumption of MED items could provide essential nutrients, including protein, vitamins, and minerals, which could contribute to maintaining muscle mass, cognitive function, and overall health in older adults. A balanced diet that included MED items could help mitigate nutritional deficiencies commonly associated with frailty, thereby slowing its progression over time [52]. Other than that, we put forward the substitution effect of moderate MED consumption: for Chinese older adults, who are more apt to light and bland diets, consuming moderate amounts of MED items may lead to a more balanced and diverse diet, which could lead to a lower intake of unhealthy foods high in saturate fats, sugars, and processed carbohydrates [53]. Such dietary patterns have been associated with a reduced risk of chronic diseases and frailty, as they may help maintain metabolic health.

Moderate intake is good but excessive consumption is bad. Contrary to expectations, it was revealed that some individuals reporting 3-MED exhibited a faster increase in frailty levels compared to those with 0-MED consumption. This unexpected finding has raised important questions about the potential diverse effects of excessive MED consumption on frailty levels among Chinese older adults. We put forward that a high consumption of MED items, especially when combined, may lead to an excessive intake of saturated fats, cholesterol, and other unhealthy components, which could contribute to the development or exacerbation of chronic diseases associated with frailty, such as cardiovascular disease, diabetes, and obesity [54,55]. Additionally, the excessive consumption of certain MED items, such as processed meats and full-fat dairy products, may also contribute to inflammation and oxidative stress [56], further accelerating frailty progression. Therefore, it is essential to consider the quality of MED items consumed, as well as overall dietary patterns when assessing their impact on overall frailty. Another explanation could be related to the dietary imbalances caused by 3-MED consumption: older adults consuming high levels of MED items may disproportionately prioritize these foods over other essential components of a healthy diet, such as fruits, vegetables, whole grains, and legumes. Such dietary imbalances could lead to deficiencies in key nutrients including fiber and antioxidants, which are essential to maintain overall health and reduce the risk of chronic diseases and frailty.

Meanwhile, the accelerated increase in frailty among rural residents consuming high levels of MED items underscored the importance of considering the rural/urban divide: rural residents, particularly those in remote areas, may have limited access to diverse food sources, including protein sources. As a result, they may rely heavily on locally available foods, and the quality and variety of MED items in rural areas may be lower, potentially leading to a higher consumption of processed and unhealthy MED products. Older adults with smaller social networks or fewer social contacts generally also had a lower-quality diet and tended to have repetitive food intake [57], potentially contributing to the unbalanced MED consumption. Moreover, the traditional dietary practices rooted in agricultural lifestyles are more common among rural residents, and older adults in rural areas may have inherited dietary habits shaped by historical contexts, including periods of food scarcity and agrarian lifestyles [58]. They are accustomed to eating up all they have and never allow any leftovers, and lack of nutritional knowledge may lead to misperceptions of “the more you eat, the healthier you’ll be” [59]. The firm belief in cumulative protein and MED consumption is broken.

Lastly, the findings indicating a faster increase in frailty among female residents has highlighted the gender-specific dynamics influencing dietary habits and health outcomes among Chinese older adults. The traditional gender roles and responsibilities in Chinese society may contribute to dietary habits and health behaviors among older adults [30]. Women, particularly in older generations, often bear the responsibility for meal preparation and family caregiving, leading to differential dietary patterns. Females may prioritize family preferences over personal dietary choices, resulting in higher consumptions of MED items, including fatty meats and dairy products, which can often be too much for them to digest. Additionally, socio-cultural expectations regarding body image and weight management may influence women’s dietary behaviors, potentially impacting their frailty risk [60,61]. Apart from that, we put forward those biological factors, including hormonal and physiological differences between men and women, which may influence the relationship between MED consumption and frailty progression. Women experience unique health challenges related to menopause, osteoporosis, and sarcopenia, which may increase their susceptibility to frailty due to declining estrogen levels, exacerbated bone loss, and muscle wasting. The over-consumption of MED products in trying to make up for the frailty could lead to the inverse effect.

This study has its limitations: one limitation of this study is the incomplete assessment of frailty measures across all waves of data collection: important indicators such as cognitive function were only available in the 2018 wave of the study. Another limitation is the reliance on self-reported data for dietary habits and frailty assessment which may induce bias and measurement errors. Lastly, the rural/urban subsamples were imbalanced, since there were a much higher proportion of rural residents in the study.

## 6. Conclusions

Despite such limitations, this longitudinal study investigating the association between MED consumption and frailty among Chinese older adults over a decade has yielded significant insights into the complex interplay between dietary habits, demographic factors, and frailty progression. The general trend of increasing frailty levels across MED consumption categories underscored the significance of dietary patterns in influencing health outcomes in aging populations. However, we also found the potential protective effect of moderate MED consumption, as well as a faster increase in frailty linking to high MED consumption, particularly among rural and female residents. These findings have important implications for public health policies and interventions aimed at promoting healthy aging in Chinese older adults. Policy suggestions include targeted nutritional education programs emphasizing balanced MED consumption and promoting traditional dietary practices that prioritize light, non-greasy foods. Additionally, interventions tailored to specific demographic groups, such as rural and female residents, should address socioeconomic disparities and cultural influences on dietary habits.

## Figures and Tables

**Table 1:** Sample characteristics at baseline (Year 2008).

	Whole (*N* = 2312)	Rural (*N* = 1717)	Urban (*N* = 595)	Male (*N* = 1085)	Female (*N* = 1227)
*Main IV*	N (%)				
MED Consumption					
0	286 (12.37)	241 (14.04)	45 (7.56) ***	101 (9.31)	185 (15.08) ***
1	745 (32.22)	620 (36.11)	125 (21.01)	347 (31.98)	398 (32.44)
2	1085 (46.93)	779 (45.37)	306 (51.43)	538 (49.59)	547 (44.58)
3	196 (8.48)	77 (4.48)	119 (20.00)	99 (9.12)	97 (7.91)
*Categorical*					
Gender: male	1085 (46.92)	796 (46.36)	289 (48.57)		
*Hukou*: rural	1717 (74.26)			796 (73.36)	921 (75.06)
Married/Partnered: Yes	1415 (61.20)	1026 (59.76)	389 (65.38) **	845 (77.88)	570 (46.45) ***
Literate: Yes	1202 (52.03)	795 (46.36)	407 (68.40) ***	807(74.38)	395 (32.24) ***
Income: sufficient	1795 (77.64)	1287 (74.96)	508 (85.38) ***	864 (79.63)	931 (75.88) **
Exercise: Yes	815 (35.25)	454 (26.44)	361 (60.67) ***	441 (40.65)	374 (30.48) ***
Smoking: Yes	525 (22.71)	406 (23.65)	119 (20.00) *	458 (42.21)	67 (5.46) ***
Drinking: Yes	517 (22.36)	405 (23.59)	112 (18.82) **	416 (38.34)	101 (8.23) ***
*Continuous*	Mean (SD)				
Age	75.41 (8.32)	75.45 (8.41)	75.32 (8.05)	74.38 (7.63)	76.31 (8.78) ***
Life satisfaction	3.62 (0.81)	3.56 (0.81)	3.77 (0.79) ***	3.66 (0.79)	3.58 (0.82) **
Psychological Well-being	2.95 (0.44)	2.93 (0.45)	2.99 (0.41) ***	2.93 (0.41)	2.96 (0.47) *
Frailty × 10	0.76 (0.07)	0.75 (0.07)	0.79 (0.07) *	0.61 (0.06)	0.89 (0.08) ***
Frailty range	0–0.60	0–0.60	0–0.47	0–0.60	0–0.46

*Note:* Unpaired *t*-tests and ANOVA tests have been carried out to compare rural/urban and gender differences. * *p* < 0.05; ** *p* < 0.01; *** *p* < 0.001.

**Table 2:** Generalized equation estimation (GEE) model results: impact of MED consumption on frailty.

	Whole Model 1	Rural Model 2	Urban Model 3	Male Model 4	Female Model 5
*Focal IV*	Estimates (SE)				
MED Consumption					
0 (reference)					
1	−0.052 (0.045) **	−0.056 (0.048) *	−0.044 (0.114) *	−0.021 (0.061) *	−0.103 (0.061) **
2	−0.010 (0.044)	−0.026 (0.047)	0.055 (0.109)	0.012 (0.059)	−0.024 (0.060) *
3	0.024 (0.054)	0.102 (0.067) *	−0.015 (0.114)	0.006 (0.071)	0.049 (0.078)
*Categorical*					
Gender: male	−0.211 (0.028) ***	−0.211 (0.031) ***	−0.224 (0.060) **		
*Hukou*: rural	−0.124 (0.028) **			−0.160 (0.038) ***	−0.093 (0.042) **
Married/partnered: Yes	−0.045 (0.026) *	−0.073 (0.028) *	0.043 (0.058)	−0.065 (0.037) *	−0.019 (0.036)
Literate: Yes	−0.043 (0.025) *	−0.084 (0.027) **	0.084 (0.058)	−0.058 (0.035) *	−0.021 (0.035)
Income: sufficient	−0.059 (0.028) *	−0.059 (0.031) *	−0.086 (0.068)	−0.101 (0.039) **	−0.031 (0.040)
Exercise: Yes	0.012 (0.023)	0.016 (0.027)	0.015 (0.048)	0.018 (0.031)	0.016(0.037)
Smoking: Yes	−0.041 (0.026)	−0.060 (0.030) *	0.016 (0.056)	−0.045 (0.028)	−0.075 (0.073)
Drinking: Yes	−0.141 (0.025) **	−0.083 (0.028) *	−0.288 (0.050) **	−0.097 (0.027) **	−0.302 (0.055) ***
*Continuous*					
Age	0.032 (0.002) ***	0.030 (0.002) ***	0.038 (0.003) ***	0.026 (0.002) ***	0.037 (0.002) ***
Life satisfaction	−0.033 (0.015) *	−0.027 (0.017)	−0.056 (0.033)	0.012 (0.021)	−0.070 (0.021) **
Psychological well-being	0.022 (0.031)	−0.016 (0.034)	0.152 (0.069) *	−0.038 (0.042)	0.059 (0.043)

Note: * *p* < 0.05; ** *p* < 0.01; *** *p* < 0.001.

## Data Availability

All data used in this study are publicly available on the CLHLS website https://opendata.pku.edu.cn/dataverse/CHADS (accessed on 1 January 2024).

## Supplementary Materials

The following supporting information can be downloaded at: https://www.mdpi.com/article/10.3390/nu16091334/s1, Table S1: Scoring Criteria for Frailty; Table S2: MED and Frailty Levels Over Four Waves.
