# Supplementary material for: Meat–Egg–Dairy Consumption and Frailty among Chinese Older Adults: Exploring Rural/Urban and Gender Differences
Source: Nutrients. 2024 Apr 28;16(9):1334. doi: 10.3390/nu16091334 (PMC11085892; doi:10.3390/nu16091334)
Supplement: Supplementary file 1 [file nutrients-16-01334-s001.zip › nutrients-2975361-supplementary.pdf]

nutrients MDPI

Table S1. Scoring Criteria for Frailty.

Variables	Cut-Off Point
1. Self-reported health	V. good = 0, good = 0.25; okay= 0.5, bad = 0.75, very bad = 1
2. Bathing	Without assistance = 0, one part assistance = 0.5, more than one part assistance = 1
3. Dressing	Without assistance = 0, one part assistance = 0.5, more than one part assistance = 1
4. Toileting	Without assistance = 0, one part assistance = 0.5, more than one part assistance = 1
5. Transferring	Without assistance = 0, one part assistance = 0.5, more than one part assistance = 1
6. Continence	Without assistance = 0, one part assistance = 0.5, more than one part assistance = 1
7. Feeding	Without assistance = 0, one part assistance = 0.5, more than one part assistance = 1
8. Hypertension	Yes = 1, no = 0
9. Diabetes	Yes = 1, no = 0
10.Heart disease	Yes = 1, no = 0
11. Stroke or CVD	Yes = 1, no = 0
12. Bronchitis et al	Yes = 1, no = 0
13. Tuberculosis	Yes = 1, no = 0
14. Cataract	Yes = 1, no = 0
15. Glaucoma	Yes = 1, no = 0
16. Gastric or Duodenal ulcer	Yes = 1, no = 0
17. Arthritis	Yes = 1, no = 0
18. Cholecystitis	Yes = 1, no = 0
19. Blood Disease	Yes = 1, no = 0
20. Nephritis	Yes = 1, no = 0
21. Visual function	Can see and distinguish (1) = 0, can see only (2) = 0.5, can't see (3) = 1, blind (4) = 1
22. Rhythm of heart	≥ 80bpm = 1;<80bpm = 0
23. Hand behind neck	Both hands (3) = 0, left hand(2) = 0.5, right hand(1) = 0.5, neither hand(4) = 1
24. Hand behind lower back	Both hands (3) = 0, left hand(2) = 0.5, right hand(1) = 0.5, neither hand(4) = 1
25. Able to stand up from sitting	Yes, without using hands (1) = 0, Yes, using hands(2) = 0.5, no (3) = 1
26. Able to pick up a book from the floor	Yes, standing (1) = 0, Yes, sitting (2) = 0.5, no (3) = 1
27. Number of serious illness past 2 years	Yes = 1, no (0) = 0
28. Able to hear	Yes = 0, need aid=0; despite aid=1, no = 1
29. Interviewer rated health	Surprisingly healthy = 0, relatively healthy = 0, moderately ill = 0.5, very ill = 1
30. Able to use chopsticks	Yes (1) = 0, no(2) = 1
31. Number of steps to turn around	≥ 6 steps = 1, <6 steps = 0
32. Able to go out	Yes=0, some difficulty=0.5, no=1
33. Able to go shopping	Yes=0, some difficulty=0.5, no=2
34. Make food by self	Yes=0, some difficulty=0.5, no=3
35. Wash clothes by self	Yes=0, some difficulty=0.5, no=4
36. Walk 1 km	Yes=0, some difficulty=0.5, no=5
37. Carry 5kg weight	Yes=0, some difficulty=0.5, no=6
38. Crouch and stand	Yes=0, some difficulty=0.5, no=7
39. Take public transport	Yes=0, some difficulty=0.5, no=8

Table S2. MED and Frailty Levels Over Four Waves.

	Wave 1 (2008)	Wave 2 (2011)	Wave 3 (2014)	Wave 4 (2018)
Whole Sample				
MED Consumption	N (%)			
0	286 (12.37)	191 (8.26)	124 (5.36)	153 (6.62)
1	745 (32.22)	715 (30.93)	670 (208.98)	622 (26.90)
2	1085 (46.93)	1171 (50.65)	1303 (56.36)	1189 (51.43)
3	196 (8.48)	235 (10.16)	215 (9.30)	348 (15.05)
Frailty	Mean			
Frailty × 10	0.76	0.93	1.08	1.67
Frailty range	0–0.60	0–0.63	0–0.68	0–0.78
Rural Subsample				
MED Consumption	N (%)			
0	241 (14.04)	151 (8.79)	101 (5.89)	125 (7.28)
1	620 (36.11)	587 (34.19)	565 (32.91)	571 (30.11)
2	779 (45.37)	885 (51.54)	963 (56.09)	872(50.78)
3	77 (4.48)	94 (5.48)	88 (5.12)	203 (11.82)
Frailty	Mean			
Frailty × 10	0.75	0.90	1.05	1.63
Frailty range	0–0.60	0–0.63	0–0.65	0–0.78
Urban Subsample				
MED Consumption	N (%)			
0	45 (7.56)	40 (6.72)	23 (3.86)	28 (4.70)
1	125 (21.01)	128 (21.51)	105 (17.65)	105 (17.65)
2	306 (51.43)	286 (4.81)	340 (57.14)	317 (53.28)
3	119 (20.00)	141 (23.70)	127 (21.34)	145 (24.37)
Frailty	Mean			
Frailty × 10	0.78	1.03	1.21	1.79
Frailty range	0–0.47	0–0.60	0–0.68	0–0.78
Male Subsample				
MED Consumption	N (%)			
0	101 (9.31)	70 (6.45)	39 (3.59)	59 (5.43)
1	347 (31.98)	297 (27.37)	290 (26.73)	266 (24.51)
2	538 (49.59)	603 (55.58)	653 (60.18)	582 (53.64)
3	99 (9.12)	115 (10.60)	103 (9.49)	178 (16.40)
Frailty	Mean			
Frailty × 10	0.61	0.76	0.89	1.42
Frailty range	0–0.60	0–0.59	0–0.68	0–0.73
Female Subsample				
MED Consumption	N (%)			
0	185 (15.08)	121 (9.86)	85 (6.92)	94 (7.66)
1	398 (32.44)	418 (34.07)	380 (30.97)	356 (29.01)
2	547 (44.58)	568 (46.29)	650 (52.97)	607 (49.47)
3	97 (7.90)	120 (9.78)	112 (9.13)	170 (13.85)
Frailty	Mean			
Frailty × 10	0.64	1.09	1.26	1.89
Frailty range	0–0.46	0–0.63	0–0.68	0–0.78
